# Post-genomic progress in helminth parasitology

**DOI:** 10.1017/S0031182020000591

**Published:** 2020-07

**Authors:** Paul McVeigh

**Affiliations:** School of Biological Sciences, Queen's University Belfast, 19 Chlorine Gardens, Belfast BT9 5DL, UK

**Keywords:** Anthelmintic resistance, CRISPR-Cas9, functional genomics, genome, helminth, proteome, RNAi, single-cell, transcriptome

## Abstract

Helminth parasitology is an important discipline, which poses often unique technical challenges. One challenge is that helminth parasites, particularly those in humans, are often difficult to obtain alive and in sufficient quantities for study; another is the challenge of studying these organisms *in vitro* – no helminth parasite life cycle has been fully recapitulated outside of a host. Arguably, the key issue retarding progress in helminth parasitology has been a lack of experimental tools and resources, certainly relative to the riches that have driven many parasitologists to adopt free-living model organisms as surrogate systems. In response to these needs, the past 10–12 years have seen the beginnings of helminth parasitology's journey into the ‘omics’ era, with the release of abundant sequencing resources, and the functional genomics tools with which to test biological hypotheses. To reflect this progress, the 2019 Autumn Symposium of the British Society for Parasitology was held in Queen's University Belfast on the topic of ‘post-genomic progress in helminth parasitology’. This issue presents examples of the current state of play in the field, while this editorial summarizes how genomic datasets and functional genomic tools have stimulated impressive recent progress in our understanding of parasite biology.

## Introduction

Helminths comprise the parasites commonly referred to as ‘worms’, from the phyla Nematoda and Platyhelminthes. These are perhaps most notable for including many parasites of humans, animals and plants, which cause neglected tropical diseases in humans (Hotez, 2018), and economic losses in our agricultural and horticultural production systems (Morgan *et al*., 2019). In general, these infections are controlled through application of one of several classes of drugs known as anthelmintics, or nematicides in the case of plant-parasitic nematodes. Anthelmintics are most commonly used in mass drug administration programmes for at-risk human communities, and in herd-level treatment of farm animals. Decades of inappropriate use of these compounds has contributed to the global distribution of anthelmintic-resistant parasites. This is most notable in economically important nematode parasites of farm animals; there are farms in the Southern Hemisphere hosting nematodes that are resistant to all available anthelmintics (Kaplan and Vidyashankar, 2012). Vaccines are available only for a couple of species (Claerebout and Geldhof, 2020), leaving anthelmintics with the major burden of helminth control. Clearly, new and improved control methods are needed for helminth parasites. This need is a major stimulus for helminth parasitology research – through enhanced understanding of parasite biology, we hope to be able to identify new ways to interfere with the survival and virulence of these pathogens. The availability of genome and associated datasets, and a range of molecular tools with which to interrogate and understand these data in the context of worm biology, are therefore all of key importance in our pursuit of new drugs, vaccines and alternative control strategies. The past decade has seen a surge in the availability of such resources, enhancing our ability to interrogate parasite biology and apply that knowledge to helminth control. This Special Issue of *Parasitology* synthesizes six invited papers associated with the British Society for Parasitology (BSP)'s 2019 Autumn Symposium, each of which focuses on applications of genomic and post-genomic tools to helminth parasitology. In this Editorial, I have highlighted a small selection of the helminth research areas that have been positively influenced by improved datasets and molecular tools.

## High quality omics datasets for helminth parasites are a relatively recent development

Prior to the current genomic era, helminths were represented in genetic databases by expressed sequence tags, and a few transcriptomes and shotgun sequence libraries (Foster *et al*., 2005). While these were a useful resource at the time, their worth was compromised by being concentrated in a handful of core species, with the majority of parasites poorly represented. *Caenorhabditis elegans*, a nematode, was the first metazoan genome to be fully sequenced (*C. elegans* Sequencing Consortium, 1998), a project from which parasitologists undoubtedly benefitted. Many adopted *C. elegans* as a surrogate system in which to interrogate parasite biology, either as a model (Hashmi *et al*., 2001; Holden-Dye and Walker, 2014), or as a heterologous system in which to express and study parasite genes (first described by Kwa *et al*., 1995). More directly, many of the tools developed in *C. elegans* to help interrogate that organism's genome [gene silencing with RNA interference (RNAi), genetic transformation with green-fluorescent protein, genome editing with CRISPR-Cas9, for example] were later applied to nematode parasites, in some cases (described below) allowing native functional biology studies in parasites, without the need for extrapolation from a non-parasitic model system. Similarly, efforts have been made to understand flatworm parasites using free-living species such as *Schmidtea* and *Dugesia*/*Girardia*, each of which are well-resourced and tractable experimental systems, although these have never been developed into full-fledged models to the extent of *C. elegans* (Collins and Newmark, 2013; Wheeler *et al*., 2015). This may be because, as is described below, *Schistosoma mansoni*, a flatworm blood fluke parasite and cause of human schistosomiasis, is an extremely tractable experimental system in its own right. Using a native parasite model overcomes the obvious key limitation of employing free-living model systems – that they are not parasitic, and are therefore of limited use in understanding native aspects of parasite biology.

The first helminth parasite genome to be sequenced was that of the filarial nematode *Brugia malayi*, one of the causes of lymphatic filariasis (Ghedin *et al*., 2007), closely followed by the root knot nematode *Meloidogyne incognita* (Abad *et al*., 2008), and the human blood flukes *S. mansoni* and *Schistosoma japonicum* (Berriman *et al*., 2009; Zhou *et al*., 2009). In the 10 years since, we now have access to high-quality genome data for 81 species of parasitic nematode, and 31 species of parasitic flatworm (WormBase ParaSite release WBPS14; Howe *et al*., 2017). These genomes have accelerated studies in each individual species, and also supported large-scale comparative approaches for identifying the evolutionary strategies common to helminth parasitism (Zarowiecki and Berriman, 2015; International Helminth Genomes Consortium, 2019). Besides representing important resources in their own right, genome datasets have additional value in supporting the interpretation of other omics datasets. The helminth post-genomic revolution has seen a confluence of expanded genomic data with improved proteomic technologies, where in a two-way relationship, genome-sequencing data have helped to improve protein sequence identification, while proteomics similarly has helped to improve genome annotations (Ruggles *et al*., 2017). Importantly, these methods have yielded enhanced understanding of helminth secretomes (i.e. the proportion of the proteome that is secreted by the parasite into the host), augmenting our understanding of helminth immunomodulatory processes and vaccinology (Sotillo *et al*., 2017). The availability of high-quality genomics datasets has not only enhanced analyses of the protein-coding genome, it has also supported discovery of non-coding (nc)RNAs including micro (mi)RNAs (Fromm *et al*., 2017; Quintana *et al*., 2017), and long non-coding (lnc)RNAs (Vasconcelos *et al*., 2017; Liao *et al*., 2018; Oliveira *et al*., 2018). Although we still have very limited functional data on ncRNAs, it seems reasonable to expect functional insights to flow from the application of functional genomics methods to these sequences.

## Functional genomics and reverse genetics – messages in the silence

Functional genomics encompasses the experimental methods that illustrate the phenotypic output of an organism's genotype. These include transcriptomics and proteomics, both of which are used to study aspects of gene expression, and reverse genetics methods, through which we can modify an organism's genome or transcriptome and measure the impact of that manipulation on its phenotype. The development of reverse genetics methodology represented one of the most notable step changes in the history of laboratory-based biological science, revolutionizing our ability to interrogate cellular and organismal functions through induction of targeted transcriptional changes. RNAi mediated by double-stranded (ds)RNA was first developed in *C. elegans* (Fire *et al*., 1998; for which Andrew Fire and Craig Mello were jointly awarded the 2006 Nobel Prize in Physiology or Medicine). RNAi is a gene-silencing method that is now widely adopted across diverse organisms. Triggered by introducing exogenous dsRNA matching the sequence of a target gene, RNAi hijacks endogenous mechanisms to trigger destruction (‘knockdown’) of target transcripts. In *C. elegans*, this knockdown is specific, can be heritable and triggers suppression of target protein (Ahringer, 2006), leading to phenotypic changes that can be measured. RNAi has been instrumental in probing the biology of *C. elegans*, to the point where genome-wide RNAi libraries, capable of knocking down essentially every *C. elegans* gene, are a freely available community resource (Kamath *et al*., 2003). While RNAi has not yet been applied at this scale in helminth parasites, it has been employed in several species with varying levels of success (Dalzell *et al*., 2012). RNAi is most well advanced in *Schistosoma* spp. blood fluke, where since first reports in 2003 (Boyle *et al*., 2003; Skelly *et al*., 2003) more than 100 publications (at the time of writing) have reported the use of RNAi in *S. mansoni* or *S. japonicum*. These include application throughout the schistosome life cycle – in eggs (Rinaldi *et al*., 2009), *in vitro*-derived intra-molluscan larvae (Boyle *et al*., 2003; Dinguirard and Yoshino, 2006; Mourão *et al*., 2009*a*, 2009*b*, 2013; Taft and Yoshino, 2011), *in-vitro*-maintained intra-mammalian larvae, *ex vivo* adult parasites (reviewed by Da'dara and Skelly, 2015) and even through intra-venous delivery of RNAi triggers to schistosomes *in vivo* (Pereira *et al*., 2008). In illustrating the application of RNAi to different life cycle stages, these studies highlight the ability to manipulate gene function throughout the many physiologically distinct developmental stages of the schistosome life cycle. This permits in-depth studies of parasite gene function, enabling exploitation of schistosome genome data towards the identification of new treatments for human schistosomiasis. Such research could contribute to the WHO's stated goal of schistosomiasis elimination as a public health issue by 2025 (Deol *et al*., 2019).

Although RNAi is a powerful technique, a key limitation is its inability to generate gain of function (‘knock-in’) alterations. This need is met by the ongoing genome editing revolution, through the application of CRISPR-Cas9 methods to helminths. CRISPR employs components of a prokaryotic adaptive immune system, including a Cas nuclease enzyme, and a guide RNA to target the enzyme's cleavage activity (Jacinto *et al*., 2020). When these components are introduced into a eukaryotic system, they can mediate exquisitely precise genome edits. The first applications of CRISPR-Cas9 technology to helminth parasites have described its use in *Strongyloides* spp. nematodes (Gang *et al*., 2017), and in the flatworms *S. mansoni* (Ittiprasert *et al*., 2019) and *Opisthorchis viverrini* (Arunsan *et al*., 2019). All three of these studies demonstrated the existence of non-homologous end joining mechanisms, enabling specific disruption and knockout of target genes. Homology-directed repair was also employed in *Strongyloides* and *Schistosoma* to introduce new genetic information *via* a repair template (Gang *et al*., 2017; Ittiprasert *et al*., 2019). This illustrates the possibility of labelling edited organisms with a marker (such as a fluorescent protein), or introducing an anthelmintic resistance selection gene. These approaches could streamline the selection and analysis of edited individuals. If taken up by the wider community, this suite of genome editing tools should revolutionize our ability to probe and interrogate the biology of helminth parasites, and to identify new control targets in these globally important pathogens.

## Anthelmintic resistance – using big data to understand a big problem

Anthelmintics are used worldwide in parasite control programmes for both human and veterinary helminths, where widespread reliance has led to a five-decade struggle against anthelmintic resistance in helminth parasites (Sangster *et al*., 2018). This is a particular problem in veterinary parasites, such that in the Southern Hemisphere it is not uncommon to find sheep/goat farms colonized by nematodes that are resistant to all available anthelmintics (Kaplan and Vidyashankar, 2012). This situation has stimulated much research interest in the mechanisms and markers of selection for anthelmintic resistance (Tinker, 2019; Kaplan, 2020). Much of the research in these areas has centred on individual drug-resistant candidate genes, selected either from potential anthelmintic targets (Gilleard, 2006) or from hypotheses around drug deactivation/efflux mechanisms (Matoušková *et al*., 2016; Whittaker *et al*., 2017). This approach has not been widely successful, probably because of the narrow and limited assumptions upon which candidate gene selection was based (Gilleard, 2006). The availability of genome data now allows genome wide approaches for studying the genetics of anthelmintic resistance, where phenotypically distinct strains can be compared across the entirety of the genome to identify distinct regions that correlate with resistance (Doyle and Cotton, 2019). These regions are known as quantitative trait loci (QTL), and resistance-associated QTL have been identified in *Haemonchus contortus* in response to benzimidazoles and monepantel (Doyle *et al*., 2019; Niciura *et al*., 2019) and to benzimidazoles in *C. elegans* (Zamanian *et al*., 2018). These studies have the potential to identify resistance-associated single-nucleotide polymorphisms within individual alleles, therefore linking genotype to phenotype. The hope for such data is that they will almost certainly improve our ability to identify drug-resistant genotypes in parasite populations, and they may also assist efforts to overcome resistance (Doyle and Cotton, 2019).

## Secreted nucleic acids in host–parasite interactions and molecular diagnostics

One promising application of post-genomic tools to diagnostics is the use of RNA sequencing to identify secretion of small non-coding RNAs by helminths. Genome data are an essential element in the identification of ncRNAs, because their discovery hinges upon accurate mapping of millions of short-sequencing reads to a high-quality genome. These mapping data are then computationally analysed for similarity with known ncRNA sequences and/or the prediction of their existence on feasible precursor sequences. Secreted small RNAs, predominantly micro (mi)RNAs, have been reported from 15 helminth species. Since miRNAs are negative regulators of gene expression, the prevailing hypothesis posits that these molecules are released by helminths to modulate the function of host cells, and the host environment, in their favour. This is a relatively newly recognized aspect of the host–parasite interface, which has traditionally been focused on helminth-secreted proteins (Siles-Lucas *et al*., 2015; Cai *et al*., 2016; Quintana *et al*., 2017). We are beginning to see the application of post-genomic tools to test this hypothesis, and indeed the latest evidence supports defined functional roles for helminth-secreted miRNAs in modulation of specific host cell functions (Lin *et al*., 2019; Liu *et al*., 2019).

Secreted miRNAs are also of obvious appeal as new molecular biomarkers for diagnosis of helminth infections. This appeal comes from the fact that as nucleic acids, they: (i) can be detected with exquisite sensitivity and specificity using polymerase chain reaction (PCR); (ii) are systemically distributed and are detectable in blood and other biofluids and (iii) are generally stable because most are encapsulated in extracellular vesicles (Quintana *et al*., 2017; Ghalenoei *et al*., 2020). Besides being useful for detecting the presence of parasite infection, dysregulation of host miRNA profiles can identify pathology – for example plasma miRNAs can quantify the extent of liver fibrosis caused by schistosomiasis (Chen *et al*., 2019). Diagnostics is one sub-field of parasitology that stands to benefit appreciably from the genomic revolution, due to the improvements in sensitivity, specificity and throughput that genomic data and tools could bring to diagnostic tests. All of the existing ‘gold standard’ tests for diagnosis of helminth parasites rely on visual identification of helminth life stages in feces, blood or tissue samples, or immunological detection of anti-parasite antibodies (McCarthy *et al*., 2012; Charlier *et al*., 2016; Gomez-Morales *et al*., 2017; Pfister and Van Doorn, 2018). These methods are largely subjective, relatively insensitive and/or are capable of detecting only mature, patent infections. Since many helminth pathologies are the result of infections by larval parasites, the next generation of diagnostic tools must aim to provide early detection of infection. Despite apparent progress in research laboratories, there has been little translation of molecular diagnostic tools into the field (Papaiakovou *et al*., 2019).

Recent years have seen the development of metabarcoding sequence analysis methods, where DNA extracted from a complex mixture is amplified by PCR for phylogenetically informative sequences, such as mitochondrial or ribosomal genes. The resulting amplicons are subjected to next-generation sequencing (NGS) analysis, followed by bioinformatic deconvolution to identify the breadth of sequences (and therefore species) present in the original sample. Metabarcoding has transformed our ability to perform non-invasive biodiversity surveys in terrestrial, freshwater and marine environments (Deiner et al., 2017), and these methods have now been adapted for the speciation of complex mixtures of parasites in fecal samples. The gold standard for the detection of gastrointestinal nematodes of sheep, cattle and goats has, for many decades, been fecal egg counting, where eggs are separated from a homogenized fecal sample by flotation in a sugar or salt solution, then quantified and speciated by microscopy. Many eggs can be speciated by experienced microscopists, but strongylids, one of the major groupings of veterinary gastrointestinal nematodes, cannot be visually distinguished to species level. The traditional solution to this has been to perform a larval development assay, since the third larval stage that develops on pasture after hatching from strongylid eggs do exhibit individual diagnostic morphologies. However, this is a laborious method that relies on highly trained and experienced personnel to perform. Helminth genome data have permitted the adaptation of metabarcoding methods for simple (and automatable) molecular differentiation of strongylid larvae following nematode collection from fecal samples. Through amplification of the ITS-2 ribosomal DNA locus using conserved nematode primer sets, and NGS of those amplicons, accurate quantification of complex parasitic nematode communities is possible. This method is termed ‘nemabiome’ sequencing (Avramenko et al., 2015), first used in the field to illustrate the differences in gut nematode composition between cattle herds in Canada and Brazil, as well as highlighting changes in nematode communities following anthelmintic treatment (Avramenko et al., 2017). Nemabiome sequencing has since been used to quantify nematode community composition in Canadian Bison (Avramenko et al., 2018), dairy cows (Scott et al., 2019*a*, *b*), UK sheep farms (Redman et al., 2019) and horses (Mitchell et al., 2019). These methods enable high-throughput, objective surveillance of nematode parasite prevalence in veterinary herds, providing molecular support for large-scale parasite prevalence surveys and parasite risk forecasting. Although not yet applied to human parasites, nemabiome sequencing could be useful for epidemiological analysis of soil-transmitted helminth populations in the developing world.

## Single-cell transcriptomics

Following the trend for ‘big data’ in biology, the transcriptomics field has now developed methods for sequencing the RNA transcriptome of every cell in a complex cell population containing thousands of individual cells (Hwang *et al*., 2018). Single-cell RNA sequencing (scRNA-Seq) generally involves the dissociation of tissues into individual cells, followed by the encapsulation of those cells into individual lipid vesicles containing sequencing reagents and unique molecular barcodes. After cell lysis, library construction and sequencing is performed, with extensive subsequent deconvolution using custom-scripted bioinformatics analyses. This approach allows the elucidation of the transcriptome of every cell in a tissue, organ or entire organism. The resulting datasets can be clustered by cell type, permitting large scale delineation of cell type-specific transcription patterns. These methods are uniquely informative for studies as diverse as developmental biology, transcriptional regulation and stem cell research (Hwang *et al*., 2018). ScRNA-Seq has been applied to large numbers of *C. elegans* cells (Cao *et al*., 2017), but has been developed to its highest potential in the acoelomate flatworms, where scRNA-Seq is useful for ‘dissection’ of cell populations, overcoming the considerable technical challenge of physically separating closely packed cells/tissues (Hahnel *et al*., 2013). Reflecting this challenge, the first studies to sequence essentially every cell in a complete metazoan organism were performed in the flatworm model system, *S. mediterranea* (Fincher *et al*., 2018; Plass *et al*., 2018). These datasets are useful for parasitologists since they can inform conserved aspects of flatworm biology, particularly those of nerve, muscle, reproductive and stem cells, all of which are key foci for flatworm parasitologists interested in parasite control. At the time of writing, two studies, both published as pre-prints, have applied scRNA-Seq to *S. mansoni*, describing 68 distinct cell populations and the majority of the adult parasite's tissue types (Wendt *et al*., 2020), and 11 cell types in the schistosomulum, the first intra-mammalian larval stage of the life cycle (Soria *et al*., 2019). These studies describe comprehensive cellular transcriptional maps for two important stages of the intra-mammalian schistosome life cycle, providing a source of hypotheses and targets for new anti-schistosomal therapeutics.

## Conclusions and future perspectives

The surge in availability of genomic tools for helminth parasites has revolutionized our ability to probe the biology of these pathogenic organisms without having to extrapolate from experiments performed in non-parasitic model systems. Genomics and transcriptomic data have provided new insights into the evolution and comparative genomics of helminth parasites, enabled new understanding of anthelmintic resistance and our ability to measure it in the field, new data on molecular interactions between parasites and hosts, and new molecular biomarkers enabling improved helminth diagnostics. We have also seen the development of transcriptomics towards the first applications of single-cell RNA sequencing in whole flatworms. Ongoing development of functional genomics tools allow us to test hypotheses by editing the genomes and transcriptomes of helminth parasites, bringing new depths of understanding around these fascinating and pathogenically important worms. Future work is set to increase the breadth of genomic data available to us, most notably through the Darwin Tree of Life sequencing project, which aims to sequence all 60 000 eukaryotic species in the UK and Ireland (www.darwintreeoflife.org/). Since many of these organisms are helminth parasites, this project will undoubtedly contribute to our understanding of helminth genomes, and support post-genomic progress in many more medically and economically important species.
